# Pharmacokinetics in Pharmacometabolomics: Towards Personalized Medication

**DOI:** 10.3390/ph16111568

**Published:** 2023-11-07

**Authors:** Jingai Jian, Donglin He, Songyan Gao, Xia Tao, Xin Dong

**Affiliations:** 1 School of Medicine, Shanghai University, Shanghai 200444, China; 1239385287@shu.edu.cn (J.J.); hedonglin@shu.edu.cn (D.H.); 2 Institute of Translational Medicine, Shanghai University, Shanghai 200444, China; sy_gaosmmu@163.com; 3 Department of Pharmacy, Changzheng Hospital, Second Military Medical University, Shanghai 200003, China

**Keywords:** pharmacometabolomics, pharmacokinetics, personalized medication, therapeutic drug monitoring

## Abstract

Indiscriminate drug administration may lead to drug therapy results with varying effects on patients, and the proposal of personalized medication can help patients to receive effective drug therapy. Conventional ways of personalized medication, such as pharmacogenomics and therapeutic drug monitoring (TDM), can only be implemented from a single perspective. The development of pharmacometabolomics provides a research method for the realization of precise drug administration, which integrates the environmental and genetic factors, and applies metabolomics technology to study how to predict different drug therapeutic responses of organisms based on baseline metabolic levels. The published research on pharmacometabolomics has achieved satisfactory results in predicting the pharmacokinetics, pharmacodynamics, and the discovery of biomarkers of drugs. Among them, the pharmacokinetics related to pharmacometabolomics are used to explore individual variability in drug metabolism from the level of metabolism of the drugs in vivo and the level of endogenous metabolite changes. By searching for relevant literature with the keyword “pharmacometabolomics” on the two major literature retrieval websites, PubMed and Web of Science, from 2006 to 2023, we reviewed articles in the field of pharmacometabolomics that incorporated pharmacokinetics into their research. This review explains the therapeutic effects of drugs on the body from the perspective of endogenous metabolites and pharmacokinetic principles, and reports the latest advances in pharmacometabolomics related to pharmacokinetics to provide research ideas and methods for advancing the implementation of personalized medication.

## 1. Introduction

**Figure 1:**
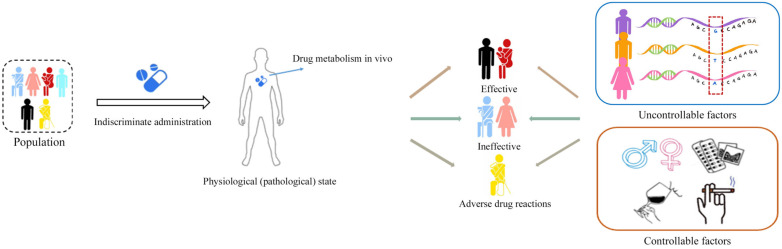
Reasons for different therapeutic responses to drugs.

The greatest difficulty encountered in drug therapy is that one drug cannot be used in a fixed dose and at the same time in an optimal regimen to treat all individuals suffering from a disease. Individual patients exhibit varying responses to the same pharmaceutical agent: some patients achieve efficacious treatment, while others show no therapeutic effects; furthermore, certain individuals may even experience adverse drug reactions (ADRs). These divergent drug responses arise as a consequence of the interplay between drug metabolism and the physiological (and pathological) state of the patient. This can also be construed as the amalgamation of the body’s disposition toward the drug and the drug’s therapeutic effect on the body. The existence of such individual diversity in the course of pharmaceutical therapy not only complicates treatment for certain patients but also poses challenges to the later stages of clinical drug trials. The origins of individual diversity predominantly stem from controllable factors, such as the patient’s lifestyle (smoking and alcohol consumption, for example), and pharmaceutical disease-related factors (the co-administration of medications and associated complications), as well as uncontrollable factors, which encompass the patient’s genetic information, age, gender, and ethnic and racial attributes [1]. Figure 1 illustrates the schematic representation of the reasons behind diverse drug responses. In response to the challenges posed by individual variability leading to complexities in drug administration, researchers have proposed the targeted approach of personalized dosing. Personalized dosing refers to tailoring monitoring or preventive strategies for a particular ailment based on a patient’s distinctive physiological condition, environmental exposures, and behavioral traits [2].

In light of the swift progress in DNA sequencing and related molecular biology methodologies, the paradigm of ‘pharmacogenomics’ emerged as a concept by researchers acknowledging the potential genetic impact on drug response phenotypes [3]. Pharmacogenomics is the study of the genomic contributions to individual differences in drug response phenotypes. Currently, research has been conducted to guide drug dosages by exploring differences in genotypes. While metformin serves as a commonly employed frontline medication in clinical diabetes treatment, notable differences in treatment responses prevail. A diabetes pharmacogenomics research team based in the United Kingdom discovered a mutation within the ATM gene (a gene involved in DNA repair and cell cycle regulation) can modify an individual’s glycemic response to metformin [4]. Henricks et al. studied dose individualization based on the DPYD genotype to reduce the toxicity induced by fluoropyrimidine and prevent deaths during the course of treatment [5]. Approximately 20% to 30% of the individual variability in drug therapy is attributed to genetic factors [6], genes play a decisive role in the upstream of life information transmission, and genetic polymorphisms can lead to differences in the activities of certain drug-metabolizing enzymes [7], drug transporter proteins [8], and drug targets [9], thereby affecting the body’s drug metabolism capabilities. Nevertheless, genetic factors can only account for a small proportion of individual variability, and the entirety of variation in drug-responsive phenotypes cannot be explained solely through genetics [10]. Organisms are defined as individuals exposed to the environment, and in addition to the “innate” ability to metabolize drugs, the individual’s lifestyle and drug-related factors have a crucial impact on the downstream, which is the ultimate expression of phenotypes in the transmission of life information. Smoking [11], age [12], and drug-drug interactions [13] all affect drug metabolism in the body to varying degrees. Metabolites serve as both intermediates and end products of biological processes, functioning as a pivotal linkage between genetic determinants and environmental influences. An organism’s metabolome comprises a diverse assembly of metabolites originating from various sources, displaying distinct types and properties [14]. It reflects the fundamental metabolic state of the organism, serving as a foundational framework for the organism’s drug clearance within a stable internal environment. The evolution of metabolomics has furnished the scientific community with technological underpinnings for the qualitative, quantitative, and dynamic characterization of metabolites. From elucidating metabolic changes associated with treatment responses to achieving individualized dosing through population stratification based on metabolic phenotypes, the prospects for the application of metabolomics are exceedingly expansive. Emerging as a novel subfield within metabolomics, pharmacometabolomics employs mathematical modeling to anticipate therapeutic responses to drugs, with it being grounded in the comprehension of individual metabolic phenotypes [15]. This approach introduces fresh research concepts and offers technical underpinnings for investigating the distinct mechanisms underlying diverse drug responses stemming from individual variability.

Furthermore, apart from investigating an organism’s drug disposition facilitated by endogenous substance metabolism, a comprehensive understanding of the drug’s intrametabolic processes within the organism is essential. Variables, including the drug’s physicochemical attributes, concentration, and dosage exert consequential influences on therapeutic outcomes. The conventional method of drug administration is to use a fixed dose and an undifferentiated administration method to guide patients to use drugs, ignoring the metabolic variability of drugs among individuals. Consequently, this approach may yield diverse therapeutic outcomes, including efficacy, inefficacy, and potentially adverse reactions. With the establishment of characterization methods for drugs in vivo, pharmacokinetics can effectively elucidate the dynamic transformations of drugs within an organism, and it can be used for the quantitative study of the absorption, distribution, metabolism, and excretion of drugs in the organism [16]. Pharmacokinetic parameters utilize a number of mathematical principles and models to illustrate more intuitively how drugs are metabolized in the body, in which the measurement of blood concentration is very indispensable for the drugs that have a narrow therapeutic window and high individual variability. The drug target needs to reach and maintain a certain concentration in order to play a role in the efficacy of the drug, and at the same time, it is also necessary to ensure that the drug concentration is within a safe range to prevent the development of toxic effects and other side effects [17]. Therefore, the pharmacokinetics of drugs in vivo is an essential aspect in our research on how to better implement personalized medication, which clearly demonstrates how drugs are metabolized in the body with the progression of time and provides a characterization method to reveal the reasons for the variability in drug delivery.

To date, there have been over 260 published research articles and reviews in the field of pharmacometabolomics, as evidenced by the results retrieved from PubMed. These publications span a range of domains, encompassing diverse drugs and diseases. The published review on pharmacometabolomics predominantly amalgamates elements of pharmacokinetics, pharmacodynamics, and the discovery of biomarkers to elucidate the relationship with precision medication. Nonetheless, specific research articles and reviews that precisely target the realm of pharmacometabolomics within the domain of pharmacokinetics have yet to be published. Building upon the foundation of metabolic regulation and drug metabolism, this review provides an overview of the evolution and utilization of pharmacometabolomics. Pharmacokinetics-related to pharmacometabolomics employs metabolomics as the analytical platform, using the baseline metabolic profile of an organism to predict inter-individual variations in drug pharmacokinetics. This approach proactively anticipates therapeutic outcomes of drugs by considering the physiological state of the organism and its environmental context. We anticipate that this approach, by synergistically considering alterations in endogenous metabolites and drug metabolism, will pave the way for novel, precision-oriented drug delivery strategies. Personalized medicine, an inevitable trajectory in future medical advancements, presents formidable implementation challenges. Confronted with diverse hurdles and lofty aspirations, alongside traditional approaches to personalized medication, there is an imperative for ongoing innovation and research endeavors. These endeavors aim to establish a robust precision drug delivery framework, enabling genuine personalization across multiple dimensions, encompassing genetics, metabolism, and pharmaceuticals. This holistic approach seeks to maximize therapeutic efficacy while mitigating the risks of adverse drug reactions and drug metabolism-related issues for patients.

## 2. Pharmacokinetics and Accurate Administration

In a systematic manner, the series of changes that occur within a biological organism following the introduction of a drug, whether stemming from the drug’s intrinsic dynamic alterations or resulting from changes in its metabolites, are challenging to observe directly. The dynamic alterations of a drug within an organism can be categorized into four processes: absorption, distribution, metabolism, and excretion, collectively forming the field of pharmacokinetics, which quantitatively investigates these processes within the biological system [18]. As a discipline grounded in mathematical modeling for analyzing the dynamic changes of drugs within biological systems, mathematical models constitute a pivotal component. The processes of drug alteration within the body are quantified using numerical representations, where parameters such as the drug concentration within the body, the drug elimination half-life, the clearance rate, and the apparent volume of distribution not only intuitively reflect the organism’s disposition to the drug but also facilitate the application of mathematical knowledge to study the underlying patterns.

The current clinical approach to implementing personalized medication is mainly therapeutic drug monitoring (TDM), which is defined as “individualizing drug dosage by maintaining plasma or blood drug concentrations within the targeted therapeutic range or window” [19]. As opposed to a fixed administered dose of a drug, blood concentrations correlate with exposure to the drug’s target and therefore better quantify the drug’s efficacy or toxicity [20]. Clinicians or pharmacists, based on the results of blood concentration measurements and pharmacokinetic principles, combined with the patient’s age, body weight, physiological (pathological) state, and other individual factors, can formulate a tailored drug regimen for this approach [21]. TDM is frequently used to monitor the following drugs: (1) drugs with no obvious pharmacodynamic indicators; (2) drugs with a narrow safety window where the therapeutic and toxic dosage ranges are in close proximity to each other; (3) drugs with poor patient compliance (medication for patients with psychiatric disorders); and (4) drugs prone to causing adverse reactions. For these drugs, their pharmacokinetics are more susceptible to variations in individual patient factors, resulting in a failure of the therapeutic response of the drug to achieve the desired effect or it even threatening the life of the patient. Consequently, there arises a necessity for the implementation of TDM. The persistent escalation of antibiotic drug resistance has been a major reason why antibiotic dosing needs to be optimized, especially for at-risk populations such as the elderly and critically ill patients [22]. Vancomycin and β-lactam antibiotics have toxic side effects; as such, the clinical use of drugs needs to be implemented for personalized medication [23]. Accurate determination of antibiotic concentrations in organisms holds paramount significance in the successful implementation of TDM, and the details of determining concentrations can exert a notable influence on the outcomes of TDM. Sofie Dhaese et al. [24] summarized the significance of monitoring the TDM of β-lactam antibiotics in critically ill patients, the targets of implementation, microbiology and sampling considerations, and proposed the limitations and solutions of the current TDM of antibiotic drugs, which holds significance in advancing the practice of tailoring antibiotic dosing guidance on an individualized basis. The TDM of antineoplastic drugs has been the focus of attention for personalized medication; this heightened focus is driven by the potential toxicity associated with antineoplastic drugs and the critical importance of monitoring clinical symptoms. Irinotecan is a topoisomerase I inhibitor used in the treatment of solid malignancies, and its therapeutic profile is characterized by a high degree of toxicity and a high degree of individual variability in pharmacokinetics [25]. Femke M de Man and colleagues conducted a comprehensive review encompassing the pharmacokinetic and pharmacodynamic characteristics of the medication, linked genetic polymorphisms with its pharmacokinetics and efficacy of irinotecan, and explored the individualized treatment of this medication from multiple perspectives [26]. As a first-line clinical antitumor drug, doxorubicin is highly effective. However, its utility is constrained by considerable toxicities and substantial pharmacokinetic variability. Despite efforts to consider recognized individual covariates, doxorubicin continues to exhibit notable pharmacokinetic variability and intricate metabolic behavior. Consequently, researchers are actively engaged in the pursuit of safer and more efficacious personalized dosing protocols for this drug [27]. Frederike K Engels and other scholars [28] designed a prospective randomized controlled trial with the aim of using AUC targeting to guide the therapeutic outcome of docetaxel administered every three weeks, with Bayesian analysis of docetaxel clearance and iterative adjustment of the dose to predict the subsequent AUC following administration. The results demonstrated that personalized medication of docetaxel based on TDM is clinically feasible and that it reduced inter-individual PK variability. TDM with pharmacokinetics as the guiding principle is an essential implementation tool to guide personalized medication. The continual advancement of technological innovations and theoretical innovations, more and more instrumentation, statistical methods and information transformation methods, all contribute to the refinement and expansion of personalized medication approaches. Nevertheless, relying solely on TDM as a single method does not achieve the goal of precise drug delivery, and TDM, in monitoring only blood concentrations, ignores the effect of individual endogenous variability on drug therapy and is not able to provide a comprehensive and accurate response to drug efficacy. We believe that it is not enough to optimize the degree of precision solely from the disposition process of the organism to the drug, but rather that there is also a need to explore the implementation of precision drug delivery from multiple perspectives in conjunction with the treatment of the organism with the drug. Relying solely on TDM and pharmacogenomics for personalized drug administration still presents certain challenges. In guiding personalized medication, it is essential to comprehensively integrate various technical approaches and data information, allowing different personalized drug administration methods to complement and validate each other. This approach facilitates more precise predictions of drug responses and monitoring of drug metabolism, advancing the implementation of personalized medication.

## 3. Endogenous Metabolites and Pharmacometabolomics

Endogenous metabolites are compounds produced by the body with a molecular weight of less than 1000 Da, such as amino acids, peptides, sugars, organic acids, lipids, vitamins, growth factors, nucleosides, and nucleotides [29]. The composition of endogenous metabolites is influenced by a variety of factors such as genomics, proteomics upstream of life information transmission, the environment in which the organism is located, diseases, and drugs. Endogenous metabolites are also an intuitive reflection and an important basis for our evaluation of the metabolic status of an organism.

With the evolution of the field of metabolomics, coupled with advancements in its analytical techniques, significant strides have been achieved in the profiling and quantification of endogenous metabolites and metabolic mechanisms in vivo, as shown by other studies [30]. Through the identification and quantification of endogenous metabolites, a number of research papers have reported and found that endogenous metabolites can influence and regulate metabolic phenotypes. For example, macrophages are an important component of the immune system and are key cells with multiple functions such as the regulation of inflammatory responses and phagocytosis; in addition, they are a class of cells with plasticity and a variety of phenotypes [31]. Shilpi Saha et al. [32] provided an extensive review elucidating the burgeoning significance of cellular metabolism and its associated metabolites as influential modulators of macrophage functionality and phenotypic attributes. Their comprehensive insights underscore the importance of comprehending the intricate interplay between metabolites, cellular function, and phenotype, which holds promise for informing subsequent investigations aimed at leveraging metabolism for the deliberate exploration of disease mechanisms and therapeutic interventions. Furthermore, metabolomics samples are more readily available compared to genomics and proteomics samples, rendering them universally applicable across a spectrum of biological research endeavors. For endogenous metabolites, drugs are also an external intervening factor of influence. As a branch in the development of metabolomics, pharmacometabolomics was born to explore the influence of environmental factors (lifestyle, environmental exposure, age, gender, drugs, etc.) in addition to genetic factors on the individual variability of drug therapy, which uses metabolomics as a technical support and drugs as the study variable to predict the therapeutic response to drugs by identifying individual metabolic characteristics represented by endogenous metabolites [33].

The formal inception of pharmacometabolomics dates back to the year 2006, marked by the pioneering work of T. Andrew Clayton and colleagues. Their seminal investigation demonstrated the composition of pre-dose urine in rats held predictive capabilities regarding the magnitude of liver injury subsequent to acetaminophen administration [34]. The general study procedure was as follows: firstly, urine samples were collected from rats before and after drug administration, respectively; four endogenous metabolites related to acetaminophen were found through 1H NMR analysis; then, the number and relative proportion of relevant acetaminophen metabolites and the degree of hepatic injury (expressed as MHS) after drug administration were determined for each rat; and histopathology and pre-dose metabolomics datasets were integrated to establish the predictive model, which was validated for stability and accuracy. In this work, not only was the concept of pharmacometabolomics demonstrated for the first time, but “Pharmacometabolomics” was defined for the first time as “the prediction of the outcome (e.g., efficacy or toxicity) of a drug or xenobiotic intervention in an individual, based on a mathematical model of ‘preintervention’ metabolite signatures”. Subsequently, the feasibility of the pharmacometabolomics concept was also validated in actual clinical samples in humans [35]. Thus, the field of pharmacometabolomics research began, wherein the principal analytical tools encompass nuclear magnetic resonance spectroscopy (NMR) and mass spectrometry (MS). NMR is a widely used analytical technique in metabolomics research. NMR emerged as the initial technology of preference for advancing the domain of pharmacometabolomics due to its exemption from the necessity for standards [36] and chromatographic separations when ascertaining the identification and quantification of target compounds [37]. Presently, it finds extensive utility in disease diagnosis, biomarker exploration for drug-induced toxicity [38,39], the prediction of metabolic profiles and PK variability of drugs [40,41], and the identification of metabolic phenotypes for effective drug response during the treatment of disease [42]. Compared with NMR, MS enjoys broader utilization in the progressive advancement of metabolomics, primarily attributable to its heightened throughput capabilities and enhanced sensitivity [43]. MS is always used as a detector coupled with chromatography, and common analytical systems include gas chromatography (GC-MS) and liquid chromatography (LC-MS). In recent years, MS-related drug metabolomics studies have covered multiple research directions [15], multiple diseases [10], multiple drugs [44], and multifaceted applications [33]. Table 1 shows the articles published from 2006 to the present day obtained by searching PubMed with the keyword “Pharmacometabolomics”, and summarizes the different diseases, drugs, research directions, and applications involved. Currently, the trajectory of pharmacometabolomics research is predominantly oriented toward pharmacokinetics, drug response (pharmacodynamics and drug toxicity), and biomarker discovery. The objects of study have also evolved from animals to healthy volunteers and then to clinical patients, and the published studies of pharmacometabolomics on animal models and human beings have involved acute and chronic diseases, including but not limited to tumors, psychiatric disorders, cardiovascular diseases, and metabolic syndrome; also, the studies on drugs have mainly focused on drugs that have a narrow therapeutic window, a wide range of individual differences in drug metabolism, an unclear target of action, as well as related mechanisms of action. Regarding its practical utility, pharmacometabolomics is primarily deployed either in parallel or in synergy with pharmacogenomics to facilitate personalized drug administration [45,46]. In addition, pharmacometabolomics is also applied during early drug development aspect [33,47], and its main role is to help to determine the safety and efficacy of drugs by predicting the drug delivery response (toxicity window and efficacy window) in conjunction with PK/PD in the late-stage clinical trial process of new drugs. In summary, pharmacometabolomics, as an emerging facet within the domain of metabolomics, has exhibited swift and substantial growth within a span of less than two decades. This expansion owes itself not only to the underpinning of sophisticated analytical methodologies but also to its prospective applicability across diverse domains within drug research. Importantly, it offers significant untapped potential in the realm of personalized drug administration, a prospect that warrants systematic examination and practical integration to contribute substantively to the ongoing advancement of human healthcare.

## 4. Pharmacometabolomics Informs Pharmacokinetics

To steer the course of personalized drug delivery, the full integration of various technical means and data information makes different personalized medication methods complement each other and demonstrate mutuality, so as to promote the implementation of personalized medication by more accurately predicting drug reactions and monitoring drug metabolism processes. A frequently employed approach for personalized drug administration in clinical settings, TDM, predominantly concentrates on the surveillance of drug metabolism (pharmacokinetics). However, it omits consideration of inter-individual metabolic variations among patients and thus falls short in its capacity to prognosticate drug response during the drug delivery process [142]. Pharmacometabolomics is the most promising method for personalized medication. Within the domain of pharmacometabolomics, its synergy with pharmacokinetics involves the utilization of metabolomics methodologies to scrutinize patients’ metabolic profiles pre- and post-drug administration. This facilitates stratification of the patient’s population using endogenous substances as biomarkers and facilitates combining the metabolism of the drug (pharmacokinetics) to predict the drug treatment response of a patient [143]. This method can simultaneously monitor the drug level (pharmacokinetics) and the patient’s individual metabolism (endogenous metabolites) level, helping to understand both the real-time metabolism of the drug in the body and the individual metabolism level of the patient through the changes in metabolic profile, which enables the doctor treating the patient to implement the clinical individualization of the patient’s medication more accurately. 

Here, we will describe in detail the experimental procedures of Pharmacokinetic-related pharmacometabolomics. In PK studies of pharmacometabolomics, researchers generally do not measure complete pharmacokinetic parameters, and the three most frequently used pharmacokinetic metrics in predicting the individualized PK of a drug or a specific pharmacokinetic parameter based on endogenous metabolites are the area under the drug time curve (AUC), the peak concentration of the drug (C_max_), and the clearance rate (CL). The AUC and C_max_ can reflect the absorption rate and degree of the drug in the body, while clearance is the apparent volume of distribution of the drug removed from the body per unit of time, elucidating the inherent disposition characteristics of the organism toward the administered drug. Especially for certain pharmaceuticals demanding heightened clinical scrutiny, the vigilant monitoring of these indices assumes paramount significance. Based on mass spectrometry technology, we established and validated a quantitative drug detection method, and blood samples at different time points after drug administration were collected to detect the blood drug concentration and related pharmacokinetic parameters with the established method, and the pharmacokinetic parameters and drug time profiles were obtained after statistical analysis.

**Figure 2:**
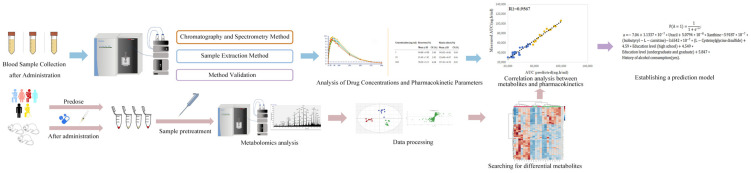
Pharmacometabolomics experimental procedures.

The metabolomics process is divided into the following steps: (a) sample collection, (b) data acquisition, (c) data processing, and statistical analysis. As shown in Figure 2.

(a) The sample collection process here mainly includes sample selection, collection, and pre-processing. Pharmacometabolomics sample selection is usually based on drug response (good or poor), and biosamples collected for analysis are typically derived from both pre- and post-drug administration time points. Urine is usually the preferred biosample for non-invasive sample collection; not only can it be collected in large quantities, but it can also characterize drug metabolism in biological organisms [144]. In Zhixin Zhang et al.’s study on the use of endogenous metabolites to predict the individualized PK of the endogenous drug bile acids, pharmacometabolomics samples were collected from pre-dose urine samples of rats for the corresponding metabolic analyses [56]. In a study on the endogenous metabolites of cytochrome P450 (CYP3A) for predicting drug drug interactions between CYP3A inhibitors and inducers in midazolam, urine samples from healthy female volunteers were also used [53]. Unfortunately, the storage conditions of urine are relatively more stringent, and it is necessary to take into account all aspects of the experimental process to achieve good results in the pretreatment of urine samples [145]. Blood is the most common bioanalytical sample for metabolomics analysis. The selection of serum and plasma, the choice of anticoagulant, and the avoidance of hemolysis are all important considerations in the analysis of blood samples [146]. Sample pretreatment is a key step in obtaining valuable metabolic information from biological samples. Based on common pretreatment methods such as protein precipitation, liquid-liquid extraction, and solid-phase extraction, selecting an appropriate extraction method according to the physicochemical properties of the analyte and the characteristics of the other matrices, and appropriately developing new techniques and methods according to the needs of the analysis, will further contribute to the utilization of metabolite analysis [147].

(b) Commonly, the analytical platforms for metabolomics are MS and NMR. As previously discussed, each of these platforms possesses distinct merits and drawbacks. In the context of pharmacometabolomics research, both MS and NMR have been utilized. However, scrutiny of Table 1 reveals, thus far, publications addressing pharmacokinetic-related drug metabolomics have not incorporated studies utilizing NMR as their analytical platform; but, the other two directions of pharmacometabolomics studies related to pharmacodynamics and biomarkers have applied NMR to their studies. Clayton [69] and others demonstrated through the use of NMR that endogenous metabolites in the urine of healthy adults could predict susceptibility to acetaminophen-induced liver injury. Hyuk Nam Kwon et al. [73] evaluated the nephrotoxicity of cisplatin using the NMR platform and predicted the toxic response based on the metabolic profile prior to administration. Table 1 shows that contemporary pharmacometabolomics investigations continue to predominantly employ the mass spectrometry (MS) platform. The MS platform can be further categorized into gas chromatography (GC-MS) and liquid chromatography (LC-MS) based on chromatographic distinctions. Chromatography-mass spectrometry is characterized by high-throughput, high-sensitivity, and rapidity and efficiency. These attributes collectively furnish essential technical underpinnings for advancing the field of pharmacometabolomics.

(c) In pharmacometabonomics experiments, variability in drug responses among patients’ post-administration can be attributed to the idiosyncrasies in their pre-administration physiological conditions, so it is typically necessary to assess the baseline metabolic profile. Raw metabolomics data obtained from analytical platforms are complex and make it difficult to acquire effective information; as such, it is necessary to use relevant analytical software to select correlated signals and reduce redundancy to generate easy-to-analyze data matrices. Taking the metabolomics data from LC-MS as an example, its pre-processing steps are generally de-noising, baseline correction, peak alignment, peak identification, and normalization [148]. Proper data preprocessing steps are an essential part of extracting important information from the raw data. In the statistical analysis of pharmacometabonomics, multivariate statistical analysis is typically used due to its capacity for investigating the influence of a multitude of variables, including pre- and post-administration, drug efficacy response, and time on the outcomes of pharmaceutical treatments [149]. Multivariate statistical analysis can be categorized into supervised and unsupervised methods, with principal component analysis (PCA) being the prevalent choice among the unsupervised techniques. In the context of metabolomics data analysis, PCA serves as the initial step, facilitating the assessment of instrument stability through the examination of quality control (QC) data distribution. Furthermore, it enables the visualization of data groupings, trends, and outliers, as well as achieving data visualization through dimensionality reduction [150]. Supervised methodologies assess the validity of classification criteria by drawing upon established sample groupings. In the field of metabolomics, widely employed techniques include partial least squares discriminant analysis (PLS-DA) and orthogonal partial least squares discriminant analysis (OPLS-DA). PLS-DA combines dimensionality reduction and regression models to perform discriminant analysis on the results using relevant discriminant criteria, which is beneficial for finding similarities and differences between multiple sets of samples. Nevertheless, a notable drawback arises when the quantity of variables surpasses the number of samples, resulting in the issue of over-fitting [151]. OPLS-DA is an extension of PLS-DA, which can better distinguish differences between groups and improve the model’s explanatory capacity while maintaining its predictive efficacy [152].

After obtaining data from both pharmacokinetic and metabolomics components, we will establish a model to evaluate and analyze the correlation between pharmacokinetic parameters and differential metabolites, and identify metabolites with predictive ability. In the corpus of published literature, the preferred approach for model construction is partial least squares (PLS) analysis. PLS analysis is a multivariate statistical data analysis method, which can quickly and efficiently screen out the relevant variables and establish the correlation between the variables, and it can be used to perform regression modeling with multiple severely correlated dependent variables. It represents a statistical analytical technique that amalgamates the strengths of principal component analysis, canonical correlation analysis, and multiple linear regression. Moreover, its response matrix has a prediction function, which is an advantageous method that meets the requirements of pharmacometabolomics data analysis. In addition, the correlation analysis of pharmacokinetic and metabolomics data will also use conventional analysis methods for calculating correlation coefficients, such as Pearman correlation coefficient and Spearman correlation coefficient [65,66,153]. Furthermore, the participation of machine learning methods in modeling also enhances the precision and efficiency of data analysis within the realm of pharmacometabolomics. The integration of machine learning with pharmacometabolomics data analysis represents a significant and valuable extension of its applicability.

## 5. Pharmacokinetics-Related to Pharmacometabolomics for Predicting Drug Reactions

Research in pharmacokinetics related to pharmacometabolomics can be categorized into specific objectives: predicting individual PK (individual metabolic characteristics of drugs), predicting the drug clearance rate, predicting the drug area under the curve (AUC) and maximum concentration (Cmax), and predicting drug exposure. Within these defined research objectives, a significant emphasis has been placed on the prediction of individual pharmacokinetics (PK) through the utilization of identified endogenous differential metabolites. Notably, groundbreaking research in this realm was conducted by Phapale et al. [48] in 2010. The researchers successfully formulated a partial least squares (PLS) model relying on the metabolic characteristics observed in pre-dose urine samples to forecast the individual pharmacokinetic profiles of tacrolimus among a diverse cohort of healthy volunteers. The study further identified key metabolites participating in the drug’s metabolism pathways, marking the initiation of using pharmacometabolomics for predicting individual PK. While the years spanning from 2010 to 2020 encompassed a significant body of pertinent studies, this review predominantly centers its attention on the academic literature from 2020 onwards, with a specific emphasis on the prediction of the individual pharmacokinetics (PK) of drugs. In 2020, Xing et al. [60] accomplished the successful prediction of individual pharmacokinetics for meropenem among a cohort of healthy volunteers by employing pre-dose plasma samples. They screened potential biomarkers to elucidate the individual variability mechanism of meropenem and validated their findings in rat experiments. This milestone signified the inaugural use of pharmacometabolomics in the prediction of individual PK profiles for an antibiotic pharmaceutical. Similarly, in 2021, this approach was applied to the context of the antidepressant medication paroxetine, which is a selective serotonin re-uptake inhibitor used to treat depression, anxiety disorders, and obsessive-compulsive disorder. Given its substantial adverse effects and a constrained therapeutic window with a narrow concentration range, the imperative for refining dosage regimens for personalized medication is underscored. In a study conducted by Zhuoling An et al. [62], an exploration was undertaken into the pharmacokinetics of paroxetine, along with the associated metabolic alterations in healthy volunteers, both pre- and post-administration. The research involved the development of partial least squares (PLS) models to establish correlations between pharmacokinetic (PK) parameters and differential metabolites. This endeavor culminated in the identification of potential biomarkers capable of predicting parameters such as the area under the curve (AUC) and maximum concentration (Cmax), thereby enabling the differentiation between individuals who respond favorably to paroxetine and those who do not. Concurrently, during the same year, attention shifted towards the cholesterol-lowering agent rosuvastatin, primarily driven by the adverse reactions it causes and the substantial inter-individual variations observed in its pharmacokinetic profiles. Anne et al. [63] studied the blood concentration and PK parameters of rosuvastatin in healthy volunteers. They analyzed pre-dose plasma metabolites using non-targeted metabolomics, correlated differential metabolites with PK parameters, and established an elastic net linear regression machine learning model, which effectively pinpointed metabolites of significance for the prediction of both the area under the curve (AUC) and the maximum concentration (Cmax) of rosuvastatin. Furthermore, there has been a significant amount of research focused on predicting drug parameters such as AUC and Cmax, which in turn predict drug exposure. In a departure from earlier investigations involving healthy human volunteers, these inquiries encompassed experimental models involving rats. Surprisingly, pharmacometabolomics was even extended to the realm of therapeutics for COVID-19, specifically in the context of the antiviral drug remdesivir. In the year 2022, Ping Du et al. [65] embarked on a pharmacometabolomics study concerning remdesivir in rat model. They employed HPLC-MS/MS to study remdesivir’s pharmacokinetic behavior and employed both cross-sectional and longitudinal metabolomics analyses. Pearson correlation analysis and PLS modeling identified potential biomarkers for predicting AUC and Cmax, which could distinguish between treatment effects. Another study from the same group in 2022 focused on the pharmacometabolomics of the tumor-targeting drug sotorasib [66], employing PK plus PM methods to predict drug exposure. In the realm of predicting drug clearance rates, a study in 2023 [68] utilized pre-dose plasma metabolomics to predict the clearance rate of busulfan, a cytotoxic drug. Through a nonlinear mixed-effects model, the population approach estimated busulfan’s clearance rate. Subsequent non-targeted metabolomics analysis of pre- and post-dose plasma samples established a linear model, which correlated the clearance rate with metabolomics results. This model could predict busulfan’s clearance rate within two weeks based on pre-dose metabolomics. Collectively, research in pharmacokinetics-related to pharmacometabolomics is experiencing robust growth. These studies underscore the versatility of pharmacometabolomics in studying drug response at various levels. This approach leverages the advantages of using endogenous metabolites to represent baseline metabolism levels and pharmacokinetics to represent drug metabolism within the body. Especially in the context of steering individualized drug dosing strategies and assessing drug safety parameters, this methodology presents a departure from the traditional belief that drug metabolism is predominantly modulated by genetic factors. It integrates additional variables such as height, weight, age, and lifestyle into drug metabolism, marking a significant advancement in medical science.

## 6. Challenges and Conclusions

With the popularization of precision medicine and the increasing enthusiasm for pharmacometabolomics research, pharmacometabolomics has been widely applied to various drugs and diseases in preclinical research. However, the translation of its findings into practical clinical applications has been sluggish, impeding the effective transference of preclinical research outcomes to the realm of precision medicine. The initial purpose of drug metabolomics was to predict the outcomes of drugs, which coincided with the most urgent problem of precision medicine. Concurrently, as pharmacometabolomics proactively anticipates individualized patient responses to drug treatments by considering patient-specific factors as variables, determining how to effectively obtain and process effective information on these baseline metabolic factors is also one of the research challenges. Although preclinical research in pharmacometabolomics has great prospects, it still requires equipment support and powerful data conversion technology to transform it into clinically applicable drug response monitoring methods. With the rise of biopharmaceuticals with strong specificity, high target selectivity, and good therapeutic effects, various malignant diseases, including tumors, can be effectively treated. Current research in pharmacometabolomics mainly focuses on small-molecule chemical drugs. Due to the large molecular weight and unique pharmacokinetic characteristics of biopharmaceuticals, there are no clear guiding principles for the characterization of the body’s disposal of biopharmaceuticals. Moreover, the drug characteristics of biopharmaceuticals, such as charge, glycosylation type, patient gene polymorphisms, and individual factor differences, have unknown effects on the ADME of biopharmaceuticals. Consequently, there is an imperative need to intensify the investigative efforts within pharmacometabolomics pertaining to the domain of biopharmaceuticals. Such endeavors are essential for refining clinical drug administration guidance and fostering innovation within the biopharmaceutical sector. In the big data environment, the reasonable utilization of artificial intelligence, large databases, and machine learning holds the potential to significantly enhance the precision and efficacy of personalized drug administration significantly. In the realm of targeted personalized therapy, the deployment of artificial intelligence and machine learning algorithms can facilitate the optimization of drug dosages. In preclinical studies of pharmacometabolomics, establishing a correct prediction model and accurately predicting PK features is a key step in data transformation. By analyzing high-throughput omics data using machine learning prediction algorithms can not only discover new biomarkers to assist in patient stratification and disease diagnosis and treatment, but also gain a deeper understanding of disease mechanisms and metabolites upstream and downstream of the pathways involved.

The development of pharmacometabolomics has only covered a period of less than 20 years, but it has led to exciting achievements. From the initial prediction of acetaminophen hepatotoxicity in rats to the current prediction of the drug toxicity of the COVID-19 drug remdesivir, pharmacometabolomics is constantly advancing through its own technological and theoretical innovation. Pharmacokinetics- related pharmacometabolomics monitors the therapeutic response of drugs from two aspects: drug metabolic behavior and changes in endogenous metabolites before and after administration and predicts the body’s response to drugs. This comprehensive approach facilitates the anticipation of an individual’s response to drugs, enabling the precise and efficient execution of personalized drug administration strategies Pharmacometabolomics provides new research ideas and methods for personalized drug delivery, but it still needs to combine technological innovation and development with other omics to achieve complementarity and mutual assistance between omics technologies. This synergy will enable a dynamic and stimulating trajectory for the evolution of drug metabolomics within the life sciences domain.

## Figures and Tables

**Table 1:** Articles published from 2006 to the present day obtained by searching PubMed with the keyword “Pharmacometabolomics”.

Pharmacokinetics-Related Pharmacometabolomics					
Number	Year	Drug	Object	Analytical Technique	Results with Pharmacometabolomics
1	2010	Tacrolimus	Healthy human volunteers	LC-MS	Predicting individualized PK of tacrolimus [48]
2	2012	Triptolide	Rats	GC-MS	Predicting the PK of Triptolide in in rats with different metabolic patterns [49]
3	2013	Midazolam	Healthy male volunteers	GC-MS	Establishing the CL equation for predicting midazolam [50]
4	2015	Atorvastatin	Healthy Volunteers	LC-MS	Predicting pharmacokinetic differences of atorvastatin in individuals [51]
5	2016	Busulfan	Allogeneic hematopoietic cell transplant recipients	LC-MS	Establishing a model for predicting the clearance rate of busulfan [52]
6	2016	Midazolam	Healthy female volunteers	LC-MS	Predicting the activity of liver CYP in women under different states [53]
7	2017	Busulfan	Paediatric haematopoietic stem cell transplantation patients	LC-MS	Potential biomarkers for predicting exposure to busulfan [54]
8	2017	Methotrexate	Patients treated with high-dose methotrexate	GC-MS	Predicting the clearance rate of methotrexate [55]
9	2017	Cholic acid	Rats	LC-MS	Using bile acid as an example to predict individualized PK [56]
10	2017	Everolimus	Heart transplant recipients	UPLC-MS/MS	Evaluating the factors affecting the metabolism of Everolimus and determine metabolic biomarkers [57]
11	2018	Zonisamide	Healthy human volunteers	LC-MS	Identification of endogenous metabolites that can predict the distribution of zonisamide [58]
12	2018	Losartan	Healthy male volunteers	NMR	Predicting individualized PK characteristics of losartan [40]
13	2018	New candidate drug	Human	LC-MS	Explaining the pharmacokinetic and pharmacodynamic characteristics of new candidate drugs [47]
14	2019	Midazolam	Healthy human volunteers	GC-MS LC-MS	Establishing an equation for predicting the clearance rate of midazolam [59]
15	2020	Faropenem	Healthy male volunteers	GC-MS LC-MS	Predicting individual PK parameters of Faropenem [60]
16	2020	Celecoxib	Healthy human volunteers	UPLC-MS/MS	Monitoring PK of celecoxib and establishing prediction models [61]
17	2021	Paroxetine	Healthy human volunteers	LC-MS	Screening and identification of endogenous markers that can predict paroxetine PK [62]
18	2021	Rosuvastatin	Healthy human volunteers	LC-MS	Predicting PK parameters of rosuvastatin [63]
19	2021	Paclitaxel	Female patients with oligometastatic breast cancer	LC-MS	Identification of pretherapeutic metabolites that may be associated with PK variability in paclitaxel [41]
20	2021	Gefitinib	Mice	UPLC-IM-MS	Analyzing the urine profile of gefitinib and analyzing PK [64]
21	2022	Remdesivir	Rats	LC-MS	Predicting AUC and Cmax of drugs [65]
22	2022	Sotorasib	Rats	LC-MS	Predicting drug exposure/toxicity biomarkers [66]
23	2022	Metformin	Healthy human volunteers	UPLC-QTOF-MS	Predicting the dose of drugs in clinical trials [67]
24	2023	Busulfan	Patients receiving HCT conditioning with Busulfan	LC-MS	Predicting the clearance rate of busulfan [68]
**Pharmacometabolomics related to drug administration response (effective, ineffective and toxic)**					
1	2006	Paracetamol	Rats	NMR	Predicting the degree of liver injury after paracetamol administration [34]
2	2009	Paracetamol	Healthy male volunteers	NMR	Determination of predictive factors for common metabolites based on urine metabolism profiles [35]
3	2010	Paracetamol	Healthy human volunteers	NMR	Identification of relevant metabolites to distinguish susceptibility to acetaminophen induced liver injury [69]
4	2011	CYP3A4 inducer	Healthy human volunteers	NMR	Predicting metabolic characteristics related to induced changes in CYP3A4 activity [70]
5	2011	3-Hydroxykynurenine	Patients with schizophrenia in first episode	LCECA	Predicting the severity of clinical symptoms in the early stages of the disease and before exposure to antipsychotic drugs [71]
6	2011	Sertraline	Patients with major depression	LCECA	Predicting whether depression patients respond to sertraline [72]
7	2011	Cisplatinum	Rats	NMR	Idiopathic and pre administration prediction of cisplatin induced nephrotoxicity [73]
8	2011	Capecitabine	Patients with colorectal cancer	1HNMR	Predicting the toxicity of capecitabine in patients with advanced colorectal cancer [74]
9	2012	Simvastatin	Healthy, first treatment drug patients	GC-MS	Identifying metabolites that can predict LDL-C responses [75]
10	2012	Galactosamine	Rats	1HNMR	Analyzing the metabolic spectrum before and after administration to understand the variable response phenotype induced by galactosamine [76]
11	2013	anti-tumor necrosis factor (ANF)	Patients with two types of arthritis	NMR	Predicting the response of patients with rheumatoid arthritis and psoriatic arthritis to TNF antagonists [77]
12	2013	Sertraline	Patients with major depression	GC-TOF	Biomarkers found to separate responders and non-responders to sertraline treatment [78]
13	2013	Sertraline	Patients with major depression	LCECA, GCTOF-MS	Distinguishing between responders and no-responders of sertraline or placebo [79]
14	2014	Aspirin	Healthy human volunteers	LC-MS	Identification of serotonin associated with aspirin response variability [80]
15	2014	Ergone	Rats	UPLC-QTOF/HDMS	Metabolic analysis of adenine induced chronic kidney disease [81]
16	2015	L-Carnitine	Septic patient	NMR	Identification of endogenous biomarkers for distinguishing between response to L-carnitine treatment in sepsis [82]
17	2015	Atenolol and Hydrochlorothiazide	Hypertensive patient	GC-MS	Identifying the characteristics of metabolites related to the treatment of two drugs and establishing predictive models [83]
18	2015	Aspirin	Healthy human volunteers	LC-MS/MS	Studying the metabolic characteristics of aspirin exposure and evaluate changes in related reactions [84]
19	2016	Atenolol	Hypertensive patient	LC-MS	The relationship between baseline serum acylcarnitine levels and cardiometabolic responses after exposure to atenolol was studied [85]
20	2016	Metformin	Non-diabetic	GC-TOF	Identification of metabolic characteristics of metformin exposure and its pharmacological effects on oral glucose tolerance [86]
21	2017	Clopidogrel	CAD patient	NMR	Identification of endogenous metabolites associated with clopidogrel HTPR in urine reveals relevant pathways and conditions [42]
22	2017	Gemcitabine	Patients with pancreatic ductal adenocarcinoma receiving gemcitabine	GC-MS	Identification of relevant differential PDAC metabolites that can predict response to gemcitabine treatment [87]
23	2017	Simvastatin	Patients treated with simvastatin	GC-MS	Predicting the risk of developing hyperglycemia or insulin resistance during simvastatin treatment [88]
24	2017	Estradiol and/or progesterone	Patients with premenstrual anxiety disorder	UPLC/MS-MS	Determining steroid-specific metabolites resulting from treatment with estradiol and/or progesterone [89]
25	2017	Cytarabine and Anthracycline	Patients with acute myeloid leukemia	UHPLC-Q-TOF	Statistical modeling of chemotherapy response in de novo AML patients treated with cytarabine and anthracyclines [90]
26	2017	Midazolam	Healthy human volunteers	GC-MS, LC-MS/MS	Validation of endogenous versus exogenous markers to assess CYP3A activity and predict treatment effects [91]
27	2017	Cisplatinum	Rats	LC-MS/MS, GC-MS	Discovery of predicted metabolites in serum prior to cisplatin administration and construction and validation of predictive models [92]
28	2018	Paclitaxel	Female adult patients with oligometastatic breast cancer	NMR	Predicting metabolic changes induced by PN and paclitaxel [39]
29	2018	Metformin	Early-stage type 2 diabetic patients	GC-MS	Predicting the efficacy of metformin [93]
30	2018	Gemcitabine-carboplatin chemotherapy	Patients with metastatic breast cancer	1H-NMR	Determining predictive metabolites for response to chemotherapy in patients with metastatic breast cancer [94]
31	2018	Dexamethasone	Rats with osteoporosis	LC-MS/MS	Predicting side effects associated with dexamethasone treatment [95]
32	2019	Dexamethasone	Preterm infants treated with dexamethasone	GC-MS	Identifying changes in metabolites before and after dexamethasone treatment can be used to distinguish between responders and no-responders [96]
33	2019	Lamotrigine and levetiracetam	Pregnant women with epilepsy	LC-HRMS	Assessing the risk of pregnant women receiving antiepileptic drug treatment [97]
34	2019	Tamoxifen	Rats	GC-MS LC-MS	Screening of potential pharmacodynamic biomarkers in rats treated with antitumor drugs under different metabolic patterns [98]
35	2019	L-Carnitine	Subjects with vasopressor-dependent septic shock treated with levocarnitine	LC-MS	Identifying differential metabolites in patients can be used to distinguish between 1-year survivors and non survivors [99]
36	2019	Irinotecan	Rats	GC-MS LC-MS	Establishing a model for predicting delayed diarrhea and CPT-11 bone marrow suppression toxicity [100]
37	2019	Isoniazide	Rats	1HNMR	Determining the variability of isoniazid toxicity reactions can be used to distinguish whether adverse reactions have occurred [101]
38	2020	Anlotinib	Terminal cancer patients	LC-MS	Exploring the utility of longitudinal pharmacometabolomics in predicting response to erlotinib in patients with nasty tumors [102]
39	2020	Meloxicam	Cats	GC-MS	Predicting adverse reactions of meloxicam [103]
40	2021	L-Carnitine	Septic patient	NMR	Different efficacy of L-carnitine found in patients with different metabolic profiles [104]
41	2021	Baoyuan decoction	Rats	UPLC-MS/MS	Analysis of endogenous metabolites associated with oral administration of Baoyuan decoction to predict PD metrics [105]
42	2022	Aspirin	Rats	NMR	Predicting gastric toxicity associated with LDA induced coronary artery disease [106]
43	2022	Angiotensin-converting enzyme inhibitors, angiotensin receptor blockers, calcium channel blockers, and diuretics	Hypertensive patient	LC-MS	Metabolic profiles based on metabolic profiles comparing metabolic profiles between four antihypertensive drug groups and non-drug groups [107]
44	2022	Gefitinib	Patients with non-small cell carcinoma	LC-MS/MS	Identification of biomarkers inducing liver toxicity [108]
45	2022	Olanzapine	Rats	AFADESI-MSI	Identification of metabolites and drug-related treatments and adverse reactions [109]
**Pharmacometabolomics related to biomarkers**					
1	2008	Paracetamol	Mice	LC-MS	Identification of biomarkers related to toxic reactions [110]
2	2008	Cisplatinum	Lung cancer patients	GC-MS	Discovering new biomarkers related to cisplatin therapy [111]
3	2008	Polychlorinated biphenyls	Mohawk men and women	LC	Validation of biomarkers based on serum metabolic profiles [112]
4	2011	Citalopram and escitalopram	Patient with major depression	GC-MS	Discovering biomarkers for citalopram/escitalopram treatment [113]
5	2012	Rifampicin	Bacterial strains	GC-MS	Comparing the fatty acid metabolites of two strains [114]
6	2013		AD subjects, mild cognitive impairment, and control	LCECA	Identify functionally relevant alterations in metabolic networks and pathways in AD [115]
7	2013		Hepatitis B virus patients	UPLC-Q-TOF-HDMS	Identifying urine biomarkers for HBV [116]
8	2013	Sparfloxacin	hamsters	LC-MS/MS	Predicting metabolic changes directly related to physiological or pathological functions and drug toxicity [117]
9	2013	Aspirin	Healthy human volunteers	GC-MS	Analyze serum samples from good and poor responders to aspirin for changes in metabolite levels [118]
10	2014	Acetaminophen	Children treated with Acetaminophen	LC-MS	Studying the association between APAP induced hepatotoxicity and long-chain acylcarnitine in children with APAP toxicity [119]
11	2014	Ketamine	People with bi-directional depression	LC-QTOF-MS	A metabolomic approach to identify potential markers of ketamine response and non-response [120]
12	2015	Acamprosate	Patients with alcohol use disorders	LC-MS	Discovering an increase in baseline serum glutamate levels as a potential biomarker associated with a positive reaction to akanic acid [121]
13	2015	Olesoxime	Patients with Amyotrophic Lateral Sclerosis	HPLC-MS/MS	Detection of metabolomic profiles of patients treated with Olesoxime and placebo and prediction modeling [122]
14	2016	Busulfan	Patients treated with Busulfan	LC-MS	Identification of Potential Other Metabolites Predicts Intravenous Leucovorin Clearance in HCT Subjects [123]
15	2016	Trastuzumab and Paclitaxel	Patients treated with trastuzumab-paclitaxel	LC-MS	Identification of biomarkers associated with trastuzumab paclitaxel therapy [124]
16	2016	Atenolol	Hypertensive patient	GC-MS	Identification of biomarkers related to glucose changes after atenolol treatment [125]
17	2016	Busulfan	Allogeneic hematopoietic cell transplant recipients	LC-MS	Identification of biomarkers predictive of leucovorin clearance by targeted drug metabolomics [52]
18	2016		Patients with liver cancer	GC–MS	Prognostic biomarkers for identifying clinical outcomes in lung cancer patients [126]
19	2017	Glimepiride	Healthy human volunteers	LC-MS/MS	Identification of endogenous metabolites affected by glimepiride administration [127]
20	2018	Clopidogrel	Patients with coronary artery disease	1H NMR	Identifying metabolic phenotypes associated with clopidogrel blood and identify relevant biomarkers [128]
21	2019		Tuberculosis patient	GCxGC-TOFMS	Determining the changes in human urine metabolome induced by TB treatment and the extent of treatment [129]
22	2019	Glucosamine antimonate	Patients with cutaneous leishmaniasis	LC-MS	Prediction and prognostic candidate biomarkers for determining the treatment outcome of meglumine antimoniate [130]
23	2020	Olanzapine	Mice	LC-MS	Identifying metabolites biomarkers in plasma associated with AP induced overeating and weight gain [131]
24	2020	Gemcitabine	Mice	LC-MS	Metabolites of potential biomarkers to identify the efficacy of gemcitabine in patients with pancreatic cancer [132]
25	2020	Warfarin	Patients treated with warfarin	NMR	Predicting INR based reactions in patients receiving warfarin treatment [133]
26	2020	Irinotecan	Cancer patients	LC-MS/MS	Detection of related metabolic changes for predicting the efficacy or toxicity of irinotecan [134]
27	2021	Busulfan	Patients receiving HCT conditioning with Busulfan	LC-MS	Identification of biomarkers related to HCT patients [135]
28	2021		Patients with high-density diffuse peritoneal carcinomatosis	LC-MS	Detection of metabolites associated with the propensity of cancer patients to experience oxidative stress and develop infections [136]
29	2021	Inhaled corticosteroids	Patient with asthma	UPLC-MS/MS	Evaluate plasma metabolomics indicators of inhaled corticosteroids to determine relevant metabolites [137]
30	2021	Gefitinib	Patient with rash victim	HPLC/MS-MS	Development of a predictive model for gefitinib-induced rash and validation of the model [138]
31	2022	Adriamycin	Mice	LC-MS	Identification of urinary biomarkers associated with sensitivity or resistance to doxorubicin [139]
32	2022	Tacrolimus	Kidney transplant patients	UPLC/Q-TOF-MS	Identifying relevant metabolites as biomarkers [140]
33	2023	Medical cannabis	Children with autism spectrum disorders	CE-TOF-MS, RRLC-TOF-MS	Determining corresponding cannabis reactivity biomarkers [141]

## Data Availability

Data sharing is not applicable.
